# The Interaction Between Timescale and Pitch Contour at Pre-attentive Processing of Frequency-Modulated Sweeps

**DOI:** 10.3389/fpsyg.2021.637289

**Published:** 2021-03-23

**Authors:** I-Hui Hsieh, Wan-Ting Yeh

**Affiliations:** Institute of Cognitive Neuroscience, National Central University, Taoyuan City, Taiwan

**Keywords:** frequency modulated sweep, mismatch negativity, pitch contour, timescale, pre-attentive processing, local, global

## Abstract

Speech comprehension across languages depends on encoding the pitch variations in frequency-modulated (FM) sweeps at different timescales and frequency ranges. While timescale and spectral contour of FM sweeps play important roles in differentiating acoustic speech units, relatively little work has been done to understand the interaction between the two acoustic dimensions at early cortical processing. An auditory oddball paradigm was employed to examine the interaction of timescale and pitch contour at pre-attentive processing of FM sweeps. Event-related potentials to frequency sweeps that vary in linguistically relevant pitch contour (fundamental frequency F0 vs. first formant frequency F1) and timescale (local vs. global) in Mandarin Chinese were recorded. Mismatch negativities (MMNs) were elicited by all types of sweep deviants. For local timescale, FM sweeps with F0 contours yielded larger MMN amplitudes than F1 contours. A reversed MMN amplitude pattern was obtained with respect to F0/F1 contours for global timescale stimuli. An interhemispheric asymmetry of MMN topography was observed corresponding to local and global-timescale contours. Falling but not rising frequency difference waveforms sweep contours elicited right hemispheric dominance. Results showed that timescale and pitch contour interacts with each other in pre-attentive auditory processing of FM sweeps. Findings suggest that FM sweeps, a type of non-speech signal, is processed at an early stage with reference to its linguistic function. That the dynamic interaction between timescale and spectral pattern is processed during early cortical processing of non-speech frequency sweep signal may be critical to facilitate speech encoding at a later stage.

## Introduction

Speech comprehension across languages depends on encoding the dynamic pitch contours of frequency-modulated (FM) sweeps over different pitch ranges and timescales. Information conveyed by the pitch variations of frequency sweeps provides the dominant cue from discriminating phonemes to interpreting sentence-level accentuations. The importance of FM sweeps in language processing has been demonstrated in numerous behavioral and neuroimaging studies. For instance, a poor auditory frequency sweep processing ability has been associated with reduced lexical tone awareness in Mandarin Chinese children (Wang et al., 2019), impaired reading skills in developmental dyslexia (Witton et al., 1998; Boets et al., 2011), and various language-based learning impairments (Tallal, 1980; Talcott et al., 2002). Cortical evidence for a link between frequency sweeps and speech encoding includes the finding that dyslexic individuals exhibit reduced neural mismatch negativities (MMNs) to FM-sweep signals (Stoodley et al., 2006). In addition to their role in speech processing, FM sweeps also play an important role in vocal emotion interpretation, music appreciation, and auditory scene analysis (Bregman, 1994; Carlyon, 1994; d'Alessandro et al., 1998; Crum and Hafter, 2008; Mampe et al., 2009).

In speech processing, the variations in the spectral contour of FM sweeps affect the interpretation of phonemes and semantic content in a language. This is especially prominent in a tonal language, such as Mandarin Chinese and Thai, where the different variations in pitch that convey meaning are mainly modulated at the fundamental frequency (F0) (Liang, 1963). In Mandarin Chinese, the four lexical tones are characterized by different F0s and frequency contours with contour variations providing the dominant cue for lexical tone recognition (Yip, 2002; Ploquin, 2013). For example, the same segmental sequence “ma” conveys four distinct meanings: “mother,” “hemp,” “horse,” and “to scold” in Mandarin Chinese when spoken with high level, high-rising, low-dipping, and high-falling contours (Ye and Connine, 1999; Tong et al., 2017). Several studies have suggested that Chinese speakers are highly dependent on F0 cues in processing lexical tones (Xu and Pfingst, 2008; Cabrera et al., 2014, 2015). In comparison, for non-tonal languages such as English, the pitch fluctuation at the first formant frequency (F1) or second formant frequency (F2) provides the dominant cue in phonemic identification (Gordon and O'Neill, 1998; Divenyi, 2009). For instance, while the phonemes /ba/ and /da/ share almost the same F0 features in their spectrograms, they can be easily distinguished based on their differential formant transitions at F2: /ba/ contains an upward frequency sweep while /da/ mainly constitutes a downward frequency sweep. Altogether, these findings indicate that F0 contours provide the dominant cue for tone recognition, whereas pitch variations conveyed by the F1 and F2 transitions provide the main cue for phonemic identification at consonant/vowel level.

The pitch contours of frequency sweeps are also modulated at different timescales to represent different levels of meanings across languages. Broadly defined, the temporal scale of frequency sweeps in spoken speech can be quantified into local and global timescales, with each commensurate with processing different information in speech and being lateralized in different hemispheres (Poeppel, 2003). In a tonal language, frequency contours that fluctuate over the local timescale (i.e., 20–40 ms) provide the dominant acoustic cues for consonant differentiation; in contrast, pitch variations over the global timescale (i.e., 100–300 ms) provide the main cues for determining different lexical meanings at the syllabic level (Poeppel, 2003; Li and Chen, 2015, 2018; Wang et al., 2019). For non-tonal languages, frequency sweeps with pitch variations at the local timescale provide the dominant cues for distinguishing phonemes. When pitch variations fluctuate at the global timescale, they are used to indicate sentence-level pragmatic semantic meanings, such as to highlight important information in the sentence (Ladd, 2008). Global-scale pitch variations are also used to analyze linguistic prosody, such as to indicate the purpose of a sentence as a question or statement (Poeppel, 2003). The emotional content of speech can also be conveyed via global timescale pitch features (Cruttenden, 1997; Yip, 2002; Gussenhoven, 2004).

Taken together, spoken and comprehending speech incorporates processing frequency-modulated acoustic information on multiple timescales (i.e., local vs. global) and frequency regions (F0, F1, F2…etc.). Specifically, tone language speakers must attend to frequency-modulated patterns at the F0 range to encode lexical contrasts for meanings, which on average occur at a longer timescale (i.e., around 150–300 ms). In comparison, frequency contours that varied at a shorter timescale (i.e., around 15–50 ms) are more germane for detecting formant transitions (F1/F2) in stop consonants and vowels. Accordingly, acoustic variations of frequency sweeps at different timescales can be encoded to signal different linguistic or paralinguistic features of spoken language depending on its spectral content. For example, it is well-known that speech perception of a voiced “ba” sound can be turned into a “ga” simply by changing the direction of the FM sweep in the third formant (Liberman et al., 1957). Changing the FM-sweep contour direction in the F0 of Mandarin tones also alters its sound and meaning. Thus, in order to follow speech effectively in verbal communication, proper neural processing of FM sweeps especially at these timescales and frequency regions must occur.

While timescale and spectral contour of frequency sweeps play important roles in differentiating speech sounds, there has been relatively little work designed to understand the interaction between the two acoustic dimensions during FM-sweep processing. Existing studies on processing lexical pitch contours have mostly focused on processing along a single dimension, either by manipulating timescales induced by lexical tone or sentence-level variations or by manipulating pitch type via contour and height differences. In general, these studies have typically employed the auditory oddball paradigm in which mismatch negativities (MMNs), a component of the event-related potentials (ERPs), are elicited to indicate the occurrence of acoustic deviants among a series of frequently occurring standard sounds. This paradigm has been widely used to investigate automatic encoding of different sound features including speech processing by human auditory cortex, eliminating task-related attention or memory confounds (Näätänen et al., 1978; Näätänen and Alho, 1997; Näätänen, 2001). Studies related to the processing of pitch timescales have demonstrated earlier MMNs to early cue divergent time point in both pitch variations induced by “Ma” at lexical-level tone and sentence-level accentuation, as well as differential MMNs in response to lexical tone and intonation-level pitch variations either within the same acoustic category or across categories (Luo et al., 2006; Ren et al., 2009; Xi et al., 2010; Yu et al., 2014; Li and Chen, 2018). Along the same line, some studies have demonstrated larger and earlier MMN response elicited by lexical tone pairs having a relatively early acoustic cue divergent point compared to later ones (i.e., T1/T2 vs. T2/T3) in Mandarin Chinese speakers (Chandrasekaran et al., 2007; Li and Chen, 2015).

On the other hand, studies that focused on investigating the effect of spectral content on pre-attentive processing of pitch contours have reported larger MMNs in response to larger contour difference in lexical tones compared to smaller ones (Chandrasekaran et al., 2007). Tsang et al. (2011) showed larger and earlier MMNs evoked by Cantonese tones with larger pitch height contrast, but that pitch contour difference influenced only the latency of P3a component. Similarly, Yu et al. (2017) used Cantonese monosyllables and showed that pitch height was processed earlier than pitch contour and further demonstrated that the extent and time course of the MMNs differ depending on whether the pitch contours consisted of phonological or acoustic information. Conjointly, these studies suggest that MMNs can be elicited either by variations in timescale or in spectral content of lexical pitch contours, leaving the interaction between these two dimensions unexamined.

This study is designed to investigate the joint impact of timescale and frequency region on perceptual and pre-attentive auditory processing of frequency sweeps, an acoustic feature critical to resolving speech sounds. Specifically, we address the following two questions: (1) Does timescale interact with spectral contour at perceptual and pre-attentive auditory processing of frequency sweep signal? (2) What is the nature of the pattern of interaction (if present) between timescale and spectral contour at early cortical processing of frequency sweeps? Is the interaction pattern in any relation to the linguistic function represented by the frequency sweep signals? We consider here two possibilities regarding this interaction based on the existing literature. One possibility is that timescale and pitch contour are processed as independent features, and thus, we should only observe MMN magnitudes comparable to the timescale of pitch variations regardless of the spectral region. An alternative possibility, however, is that the timescale of pitch variations interacts with its spectral content in some way to reflect its linguistic function which can be reflected by the amplitude or time course of the MMNs. In line with this possibility, Zatorre and Gandour's (2008) integrated account on speech processing has proposed that the brain's response to low-level acoustic features is modulated by the linguistic status of the input. Various studies using simple acoustic features such as noise bands modulated at different rates or non-speech tone sweeps emulating certain speech cues have obtained asymmetrical neural responses of auditory cortices identical to those elicited by speech sounds (Joanisse and Gati, 2003; Zaehle et al., 2004; Boemio et al., 2005; Schönwiesner et al., 2005). Accordingly, frequency-modulated sweeps with spectral and temporal properties simulating speech sounds could potentially elicit patterns of neural responses that reflect the nature of the speech information represented by these FM features.

To address these questions, we used the MMN paradigm, a widely used paradigm in the study of tonal pitch processing, to examine the processing of frequency sweeps with pitch variations fluctuating in speech-relevant timescales and spectral regions. The spectral content of frequency sweep stimuli is chosen to cover an equivalent octave range (i.e., 0.5 octave) while having the frequency span comparable to the fundamental frequency (F0) and first formant (F1) ranges in speech sounds (i.e., 180–270 and 600–900 Hz). Specifically, frequency sweeps that simulate T2 (rising) and T4 (falling) Mandarin lexical tone contours with equivalent frequency span but differ in pitch height (i.e., F0 vs. F1) and varied at different timescales (i.e., local vs. global) are employed in the present study. Based on findings from previous studies, we expect that all types of deviant frequency sweep stimuli would elicit MMNs. Further, if spectral contour interacts with timescale during pre-attentive processing of frequency sweeps, we expect to observe interactive effects between spectral contour and timescale on the MMN mean amplitudes and/or peak latencies. More specifically, if F0 contours are associated primarily with the role of detecting longer time global-scale pitch patterns and F1 contours are more germane for encoding shorter time local pitch pattern, then we expect differentiable patterns of MMN amplitude and/or peak latency to be observed accordingly.

## Materials and Methods

### Participants

Fifteen native speakers of Mandarin Chinese (six males) aged from 19 to 25 years old (mean ± SD = 22.4 ± 2.44 years) with normal hearing (self-reported) participated in the present study. All participants were right-handed as identified using the Edinburgh Handedness Inventory (Oldfield, 1971) and reported no history of neurological or psychiatric disease. No participants had ever studied abroad and the only English experiences they had were through compulsory English courses (i.e., 2 h of English class per week) taken during junior high to high school years (mean ± SD = 7.13 ± 1.25 years). Written informed consent was obtained from each participant in accordance with the Institutional Review Board at National Taiwan University, Taiwan. All procedures were approved and performed in accordance with the guidelines of the Institutional Review Board at National Taiwan University, Taiwan.

### Stimuli

**Figure 1:**
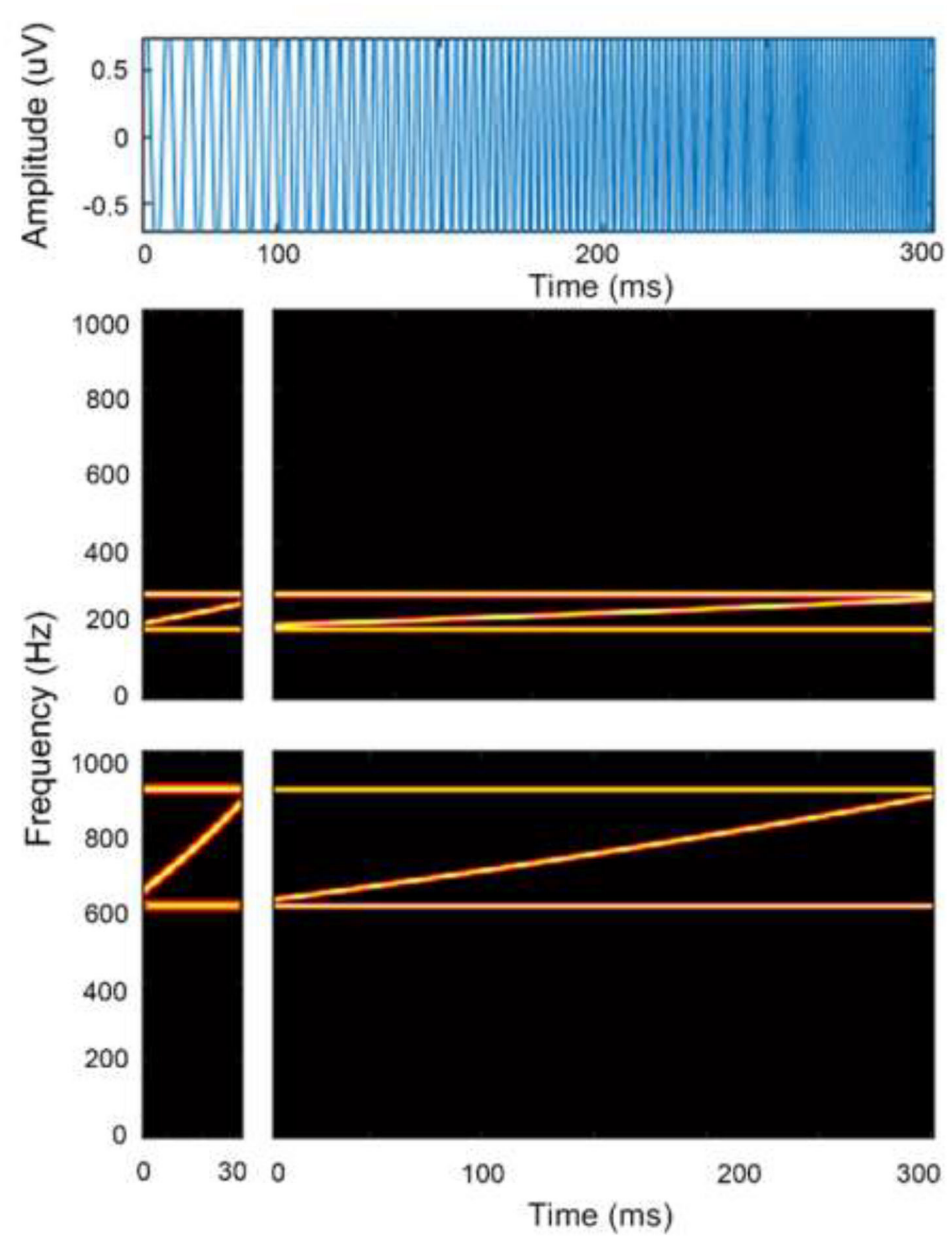
Schematic diagram of the stimulus time waveform and spectrogram of a frequency sweep. Top panel shows time waveform in blue. Bottom panels show spectrograms of a rising frequency sweep contours in the F0 (top panels) and F1 frequency (bottom panels) region at local (left panels) and global timescale (right panels). Horizontal lines in red depict bounding tones.

Stimuli were generated using MATLAB software (Matlab, 2020) on an ASUS Vento PC and presented at a rate of 44.1 kHz through 16-bit digital-to-analog converters (Creative Sound Blaster X-Fi Titanium). Sounds were presented through Sennheiser headphones (HD 380 Pro) in a double-walled, steel, acoustically isolated chamber (interior dimensions 2.5 × 2.5 × 2 m; Industrial Acoustics Company). The stimuli consisted of unidirectional linear frequency sweep complexes bounded by two steady-state pure tones. Frequency sweep was generated using the following equation:

(1) $$\begin{array}{l} X\left(t\right)=\sin\left(2\pi f_{s}\left(t\right)+\pi \frac{f_{e}-f_{s}}{T_{s}}{\left(t\right)}^{2}\right) \end{array}$$

where *f*_*s*_ and *f*_*e*_ represent the starting and ending sweep frequencies, respectively, in hertz, and *T*_*s*_ and *t* are the stimulus duration and time in seconds, respectively. A diagram of the FM-sweep stimuli waveform and spectrogram is shown in Figure 1.

There were 12 stimulus conditions (2 × 3 × 2 design): two sweep contour directions (UP or DOWN in frequency) at three stimulus durations [dur = 30, 100, or 300 ms corresponding to local (30 ms) and global (100 or 300 ms) timescales, respectively] and two F0/F1 frequency regions (F0: 180–270 Hz and F1: 600–900 Hz). The frequency bandwidth of all frequency sweep stimuli was fixed at half an octave to approximate the bandwidth of formant transitions (Luo et al., 2007). The extent and rate of these sweeps were in the general range of those observed for formant transitions and frequency glides in speech (Kewley-Port, 1982) as well as within the range used in most psychophysical and neuroimaging studies of directional FM sweeps (Poeppel et al., 2004; Brechmann and Scheich, 2005; Luo et al., 2007; Hsieh and Saberi, 2010; Kung et al., 2020). The FM complex was bounded by fixed-frequency tones, designed specifically to maintain a constant bandwidth and band position throughout the stimulus duration regardless of sweep direction (Hsieh et al., 2012). Two boundary tones were added to the frequency sweep to eliminate cues related to a shift in the centroid of spectral energy between the start and end points of the stimuli. These two boundary components were pure tones at the lowest frequency component (i.e., F0: 180 Hz and F1: 600 Hz) and the other boundary tone started at the highest frequency component (F0: 270 Hz and F1: 900 Hz); both tones remained at that frequency for the duration of the stimulus. To eliminate energy cues associated with summing of boundary tones with the frequency sweep component nearest to that tone at the point where they merged, the amplitudes of the lowest and highest FM components of the complex, as well as the boundary tones, were attenuated by 6 dB (half amplitude) using a logarithmic ramp as the frequency of the FM component approached that of the boundary tone. All stimuli were imposed with a 2-ms linear rise-and-fall time off to minimize spectral splatter and were presented binaurally via headphones (Sennheiser HD 380 Pro) at a comfortable loudness level at 70 dB SPL. Stimulus timing was controlled by voice box software implemented in MATLAB (http://www.ee.ic.ac.uk/hp/staff/dmb/voicebox/voicebox.~html).

### Procedure

Each participant was tested individually in a soundproofed shielded room. The participant was fitted with an elastic electrode cap during the electroencephalogram (EEG) part of the study. Throughout the experiment, each participant's EEG responses were continuously recorded while they watched a silent movie. The stimuli consisted of a sequence of repetitively presented standard tones that were interspersed by infrequent deviant tones, with an inter-stimulus interval (ISI) of 600 ms. The stimuli were pseudo-randomized within a block with the constraints that there were at least two standard tones between two deviant tones, and each block started with 20 presentations of the standard stimuli. For each of the stimulus conditions, there were a total of 1,000 trials per condition, consisting of 20% deviant stimuli and 80% standard stimuli.

Twelve different conditions [3 durations × 2 frequencies (F0, F1) × 2 directions] were tested in separate blocks. In each of the conditions, the direction of the tone sweep was the only difference between the deviant and the standard stimuli. For example, in the block of the DOWN 30-ms F0 condition, the standard stimulus was the 30-ms DOWN sweep and the 30-ms UP sweep served as the deviant. Hence, there were a total of 12 blocks. For each participant, the presentation order of the blocks was randomized. There were 12 blocks of 1,000 trials per block. The total recording time was ~2 h and the experiment was implemented on two separate days.

Following the EEG recordings, participants then completed the FM sweep behavioral task. We chose to have participants perform the behavioral task *after* the completion of the EEG recordings to prevent any possible bias to sweep direction caused by attentively judging the FM tone complex. The behavioral tasks consisted of a FM direction identification task in a single interval, two alternative forced choice (2AFC) paradigm. The participant is instructed to judge the direction of the sweep as UP or DOWN in frequency by pressing one of two labeled keys on the keyboard. The same 12 FM stimulus conditions (2 directions × 3 durations × 2 frequency ranges) used in the EEG study were tested. Both upward and downward FM sweeps at three different rates (30, 100, and 300 ms) were presented randomly in each of two formant frequency regions (F0 and F1). There were 600 trials in each of the two frequency ranges tested, giving a total of 1,200 trials for each participant. The inter-trial interval was 600 ms. The experiment was run in a block design in which the frequency range served as separate blocks. After half of the session, key reversal was used to eliminate key press bias. No feedback was given to the participant. All stimuli were presented binaurally through headphones at 70 dB SPL. The behavioral task took 100 min to complete.

### EEG Recording

Each participant was fitted with a Neuroscan Quik-Cap with locations of electrodes (Fz). Continuous EEG was recorded on a SynAmps2 Neuroscan system (Neuroscan, Inc., El Paso, Texas, USA) at a sampling rate of 500 Hz with a bandpass of 0.05–70 Hz (3-dB points). EEG data were recorded from 32 electrode locations in accordance with the International 10-20 Systems plus the left and right mastoid referenced to the nose tip. Horizontal electrooculogram (EOG) electrodes were placed on the outer canthi of both eyes, and the vertical EOG electrodes were placed above and below the left eye. The inter-electrode impedance was kept below 5 kΩ at all sites. Sequences were presented over headphones while participants watched a silent movie with no subtitles. Participants were seated at a viewing distance of 65 cm away from the computer monitor and were asked to focus their attention on the movie.

### EEG Data Preprocessing

EEG signal processing and analysis were carried out in MATLAB using the open-source EEGLAB toolbox (Delorme and Makeig, 2004). For each condition, the data were segmented into epochs of 500 ms, including a 100-ms pre-stimulus interval that was time locked to the onset of the auditory FM-sweep stimulus. The epochs were low-pass filtered with a cutoff frequency of 30 Hz. All the epochs were baseline corrected to the pre-stimulus interval and were subjected to the independent component analysis (ICA) to remove the components of eye movements. Epochs that contained signals exceeding a threshold of 75 μV in the EOG channels or 150 μV in the EEG channels were excluded with the threshold-method artifact rejection supplied by the EEGLAB. For each individual, the standard and deviant ERPs were automatically rejected for analysis when the trial contained root mean squared (RMS) amplitudes, computed as the square root of the mean of the squared values in the epoch, exceeding 3 standard deviations from the mean of the RMS amplitudes of all trials. On average, <20% of epochs in total were rejected. Standard and deviant ERPs were separately averaged for each stimulus condition.

### Traditional MMN Calculations

Traditional MMN waveforms were calculated by subtracting the ERP response to the up-sweep standard from the response to the down-sweep deviant stimulus in the same block. ERP and difference waves were calculated from recordings at the midline frontal site Fz, commonly suggested to generate the largest MMN response (Näätänen et al., 2007). The peak latency of the MMN component for each condition was determined for individual participants. The peak MMN latency was defined as the time point with the maximum grand average ERP amplitude at Fz electrode within a temporal window of 100–250 ms post-stimulus onset. The MMN magnitude was determined by first locating the peak amplitude of each subject within a pre-determined difference waveform window at 100–250 ms. Then, the mean MMN amplitude was computed as the mean value in the 40-ms time window centered at the peak latency of the grand average MMN waveforms (Shalgi and Deouell, 2007). This method has been suggested to be less contaminated by extreme values from each subject compared to that of the peak MMN magnitude (Luck, 2005). To compare the amplitude and peak latency of the MMNs elicited by different sweep feature conditions, two separate repeated-measures analysis of variance (ANOVA) were performed on mean MMN amplitude/latency within a specific time window at Fz with direction, formant frequency, and duration as factors.

### Same-Stimulus MMN Calculations

**Figure 2:**
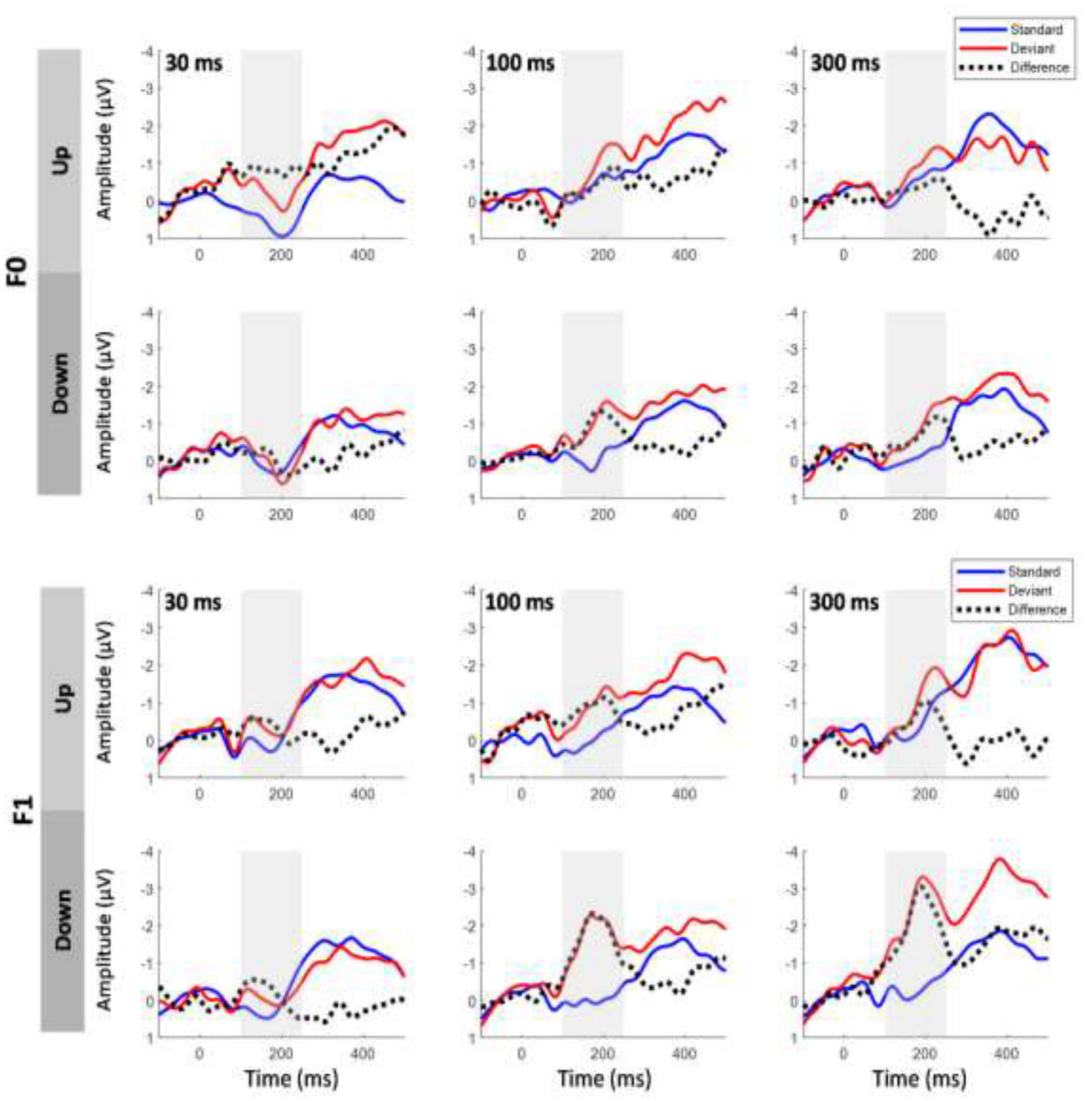
Grand average event-related potential (ERP) waveforms evoked by the standard and deviant stimuli and the difference waveforms at electrode Fz based on same-stimulus computation. Top six panels show upward and downward sweeps across all three timescales for F0 contours, and bottom six panels show the same conditions for F1 contours. Gray shaded area indicates mismatch negativity (MMN) time window. Significant MMNs were elicited in both sweep directions across all durations at the F0 and F1 frequencies.

The same-stimulus method was used to compute the difference between the ERP responses to the same stimulus presented as a standard and deviant stimulus (Pulvermuller et al., 2006; Chandrasekaran et al., 2007). In this method, the difference wave was formed by subtracting the stimulus that served as the deviant condition from the same stimulus that served as the standard condition in another block to minimize the difference of acoustical features between standard and deviant sounds. For example, the difference waveform for a 30-ms UP sweep in the F0 condition was subtracted from the ERP waveform elicited from the block in which a 30-ms UP sweep served as the standard from the block where a 30-ms UP sweep served as the deviant (i.e., 30-ms DOWN sweep block). The MMN magnitude was determined by first locating the peak amplitude of each subject within a pre-determined difference waveform window at 100–250 ms. Then, the mean MMN amplitude was computed as the mean value in the 40-ms time window centered at the peak latency of the grand average MMN waveforms (Shalgi and Deouell, 2007). Figure 2 shows overlays of the average standard (blue line) and deviant (red line) waveforms for each sweep duration condition for the F0 (top panels) and F1 (bottom panels) pitch contour conditions, respectively. The difference waveforms are shown in black dashed lines in these figures. To assess whether the two computation methods yield different MMN magnitudes, a three-way repeated measures ANOVA was performed on mean MMN amplitudes with the factors method of calculation, frequency, and duration (for comparison of the traditional and same-stimulus method for MMN computation, please refer to **Figure 5**).

### Lateralization of MMN Response

To examine whether there is an interhemispheric asymmetry of MMN response elicited by sweeps with short and long temporal scales, the individual ERPs were averaged over two clusters of the frontal central electrodes selected in each hemisphere. For the left hemisphere, the FP1, F3, F7, FC3, FT7, C3, and T3 channels were included, whereas the FP2, F4, F8, FC4, FT8, C4, and TC channels were selected for the right hemisphere. The individual MMN waveforms for each electrode cluster were computed using the same-stimulus method described above. The mean MMN amplitude computed in the 40-ms time window was entered into a three-way repeated-measures ANOVA with hemisphere, duration, and direction as factors. F0 and F1 frequencies were combined for this analysis.

## Results

Overlays of the average standard and deviant ERP waveforms and difference waves elicited by tone sweeps as a function of direction and duration in the F0 and F1 frequency regions are shown in Figure 2. We first determined whether a significant MMN response was elicited by conducting *t*-tests to compare the MMN mean amplitudes with zero values for each of the FM sweep conditions. The amplitudes of the MMN response reached statistical significance for upward tone sweeps across all durations at the F0 frequency, *t*_(14)_ = −4.018, *p* < 0.01; *t*_(14)_ = −2.554, *p* < 0.05; and *t*_(14)_ = −2.775, *p* < 0.05 for 30, 100, and 300 ms, respectively. For downward tone sweeps, the MMN amplitudes were significant across all three durations, *t*_(14)_ = −2.730, *p* = 0.016; *t*_(14)_ = −5.49, *p* < 0.001; and *t*_(14)_ = −5.038, *p* < 0.001 for 30, 100, and 300 ms, respectively. Similar patterns were observed for tone sweeps modulated at the F1 formant frequency, and all six sweep conditions also elicited significant MMN responses [all *t*_(14)_ > −3.982, *p* < 0.001].

### MMN Mean Amplitude: Effects of Sweep Features

**Figure 3:**
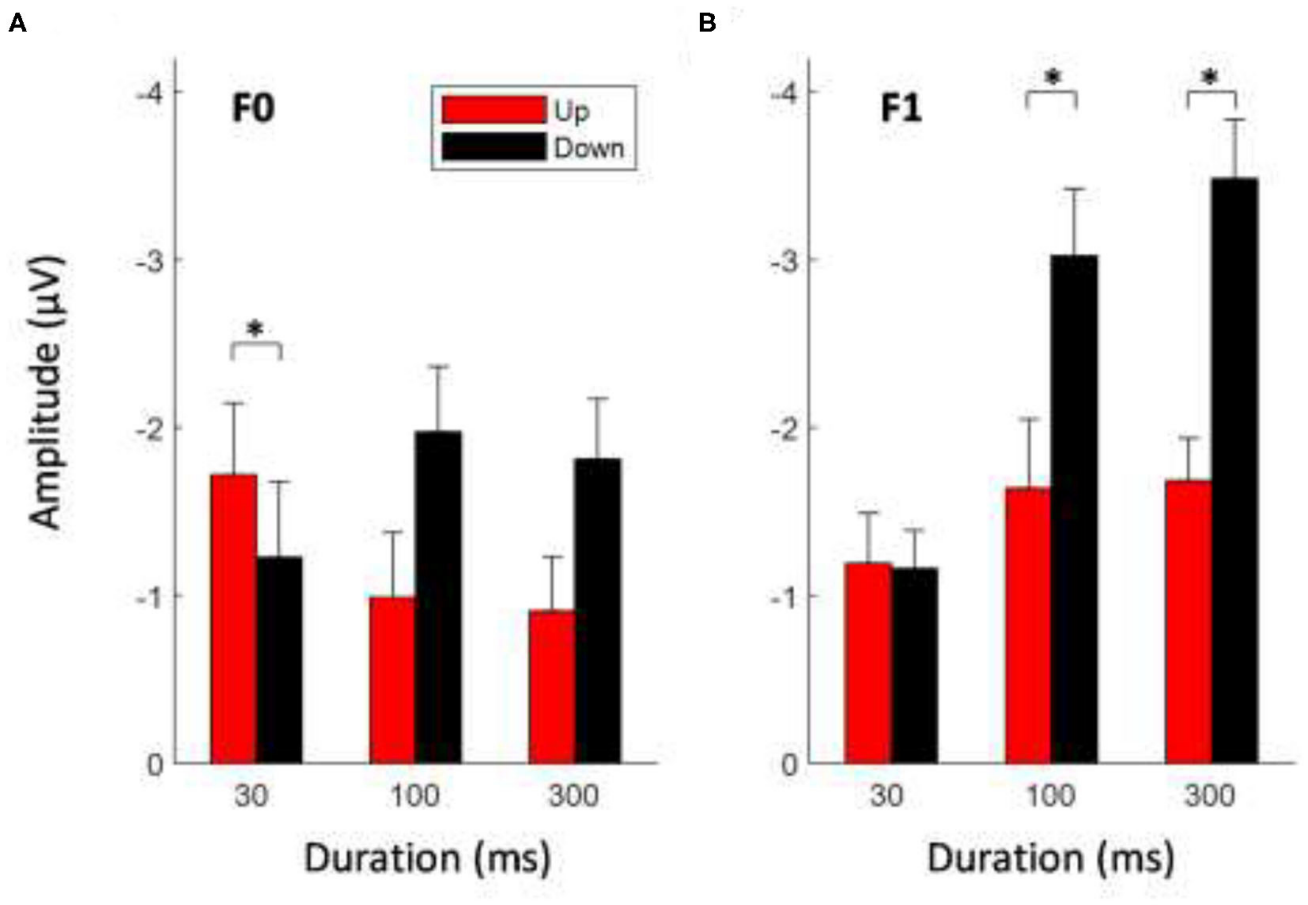
Average MMN mean amplitudes elicited by tone sweeps at different direction and duration for F0 **(A)** and F1 **(B)** frequency formant. Error bars indicate ± 1 standard error. *Indicates statistically significant at *p* < 0.05.

To determine whether the mean MMN magnitude was modulated by the sweep features, a 2 × 2 × 3 repeated-measures ANOVA was conducted on the mean MMN amplitudes at electrode Fz with the factors direction (down vs. up), formant frequency (F0 vs. F1), and duration (30, 100, and 300 ms). Figure 3 shows the mean MMN amplitudes elicited by FM sweeps at different durations and directions for F0 (Figure 3A) and F1 (Figure 3B) conditions. The analysis yielded a significant main effect of direction, *F*_(1,14)_ = 14.461, *p* < 0.01. Downward-sweeping tones elicited a significantly larger MMN response than that of upward-sweeping tones. A main effect of the frequency formant on MMN peak amplitude also reached significance, *F*_(1,14)_ = 12.30, *p* < 0.01. Frequency sweeps with pitch trajectories in the F1 frequency range (i.e., 600–900 Hz) elicited a larger MMN response than those with F0 frequency pitch contour (i.e., 180–70 Hz). In contrast, a main effect of sweep duration failed to reach significance, *F*_(2,28)_ = 3.027, *p* = 0.083. Apart from the main effects, the analysis revealed a strong three-way interaction (direction × formant frequency × duration) for MMN mean amplitudes, *F*_(2,28)_ = 6.341, *p* < 0.01 (for detailed *post-hoc* analysis on the three-way interaction, please refer to Supplementary File 1). A significant interaction between sweep duration and formant frequency on MMN response was found, *F*_(2,28)_ = 6.45, *p* < 0.05. In particular, the magnitude of the MMN response increased with increasing sweep duration at the F1 frequency range, but not for the F0 fundamental frequency range. There was also a significant interaction between sweep duration and direction on MMN amplitude, *F*_(2,28)_ = 6.156, *p* < 0.05. *Post-hoc* tests using Bonferroni corrections (family wise α = 0.05) indicated that for longer duration frequency sweeps, downward sweeps elicited a larger MMN response than during upward sweeps for both 100-ms sweeps [*t*_(29)_ = 3.267, *p* < 0.01] and 300-ms sweeps [*t*_(29)_ = 3.496, *p* < 0.01]. However, the 30-ms sweep did not elicit a different MMN response with respect to sweep direction.

### MMN Mean Amplitude: Effects of Formant Frequency

**Figure 4:**
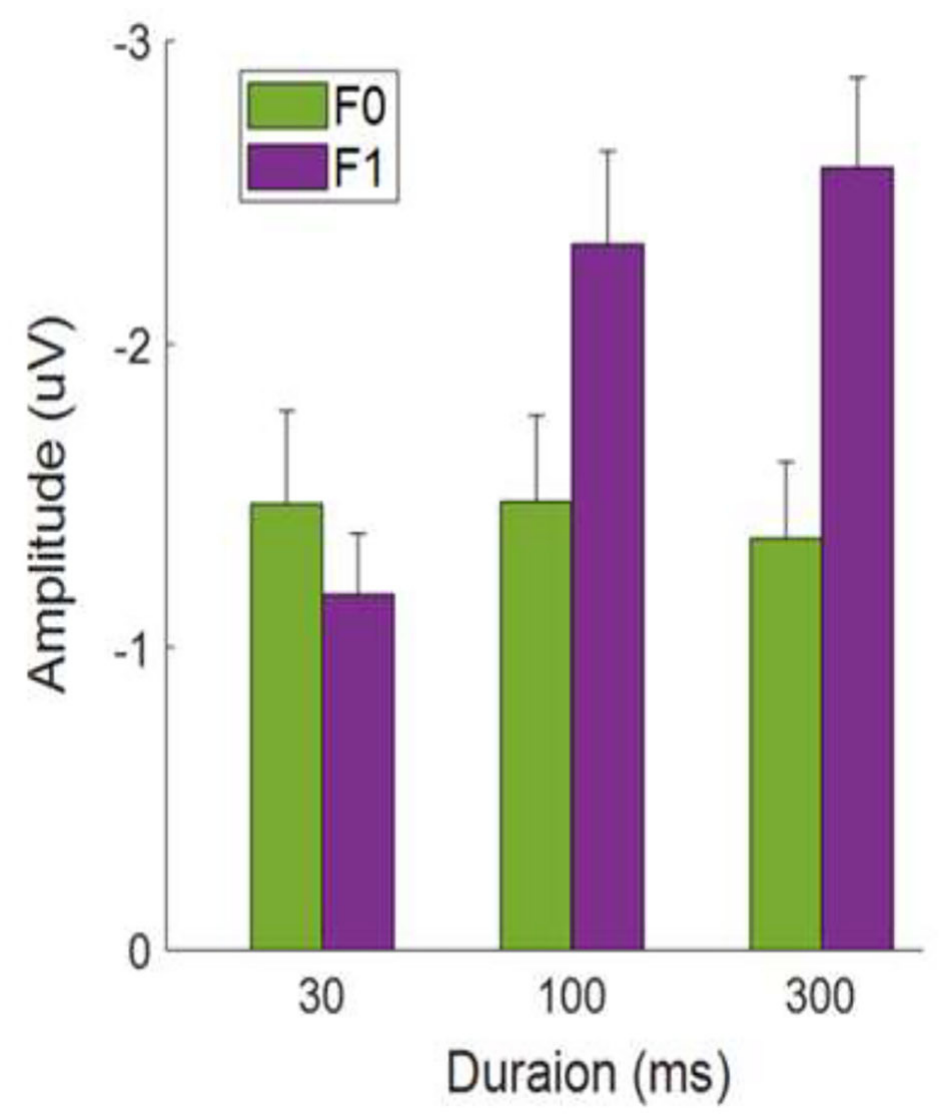
Average MMN mean amplitudes as a function of tone sweep duration. Note that up and down sweeps are combined. Error bars indicate ±1 standard error.

Figure 4 shows the average MMN amplitudes for tone sweeps at the F0 and F1 frequency regions as a function of temporal scales combined over both sweep directions. At a short (local) timescale (i.e., 30 ms), tone sweeps with F0 frequency contours elicited a larger MMN response than that of sweeps with F1 pitch contours, *t*_(29)_ = 2.035, *p* < 0.05. At a longer (global) timescale (i.e., 300 ms), this pattern was reversed; tone sweeps at the F1 frequency formant elicited a larger MMN response than that of sweeps at the F0 frequency formant, *t*_(29)_ = 3.802, *p* < 0.01.

### Comparison of Same-Stimulus and Traditional Methods

**Figure 5:**
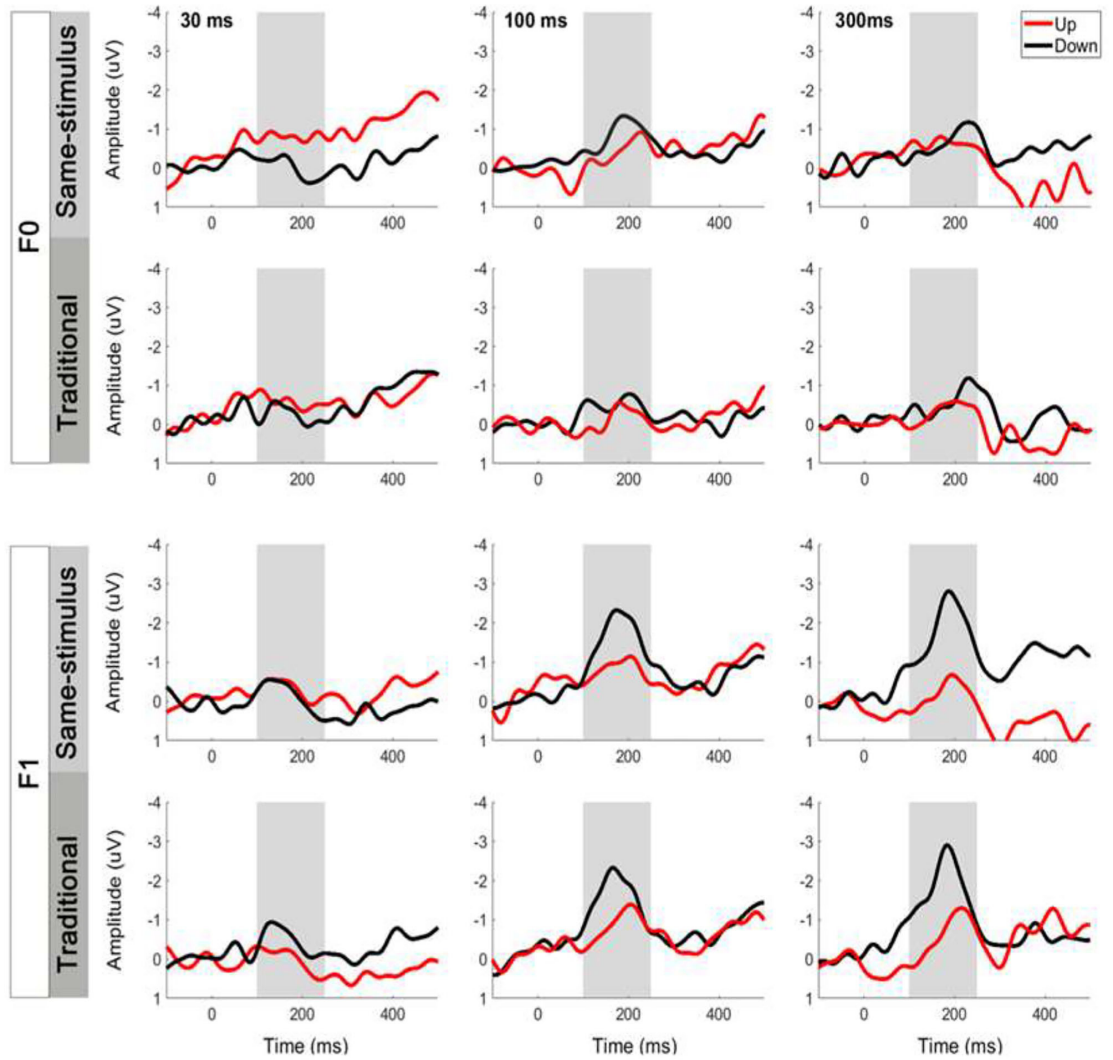
Comparison of same-stimulus and traditional methods on MMN waveforms at electrode Fz elicited by tone sweeps. Top panels show MMNs computed by same-stimulus method and bottom panels show traditional method. Gray shaded areas indicate MMN time window.

Figure 5 illustrates the average MMN waveforms elicited by upward and downward sweeps for each sweep duration calculated using the same-stimulus (top panels) and traditional (bottom panels) methods, respectively. A three-way repeated-measure ANOVA was performed with the factors of frequency (F0 vs. F1), duration (30, 100, and 300 ms), and method of calculation (same-stimulus vs. traditional) on mean MMN peak amplitudes. Upward and downward sweep responses were combined for this analysis. The ANOVA revealed a significant main effect of the tone sweep's formant frequency on the mean amplitude of the MMN response. Specifically, tone sweep modulated at the F1 formant frequency elicited a larger MMN response than that of the F0 frequency, *F*_(1,28)_ = 10.644, *p* < 0.01. There was a significant effect of tone sweep duration on mean MMN amplitude, *F*_(1,28)_ = 4.614, *p* < 0.05. The effect of the method of calculation on MMN amplitude was not significant, *F*_(1,28)_ = 0.172, *p* = 0.681. The analysis resulted in only one significant interaction, namely, between the tone sweep's formant frequency and duration, *F*_(2,28)_ = 9.511, *p* < 0.01. Specifically, F0 sweep contours evoked larger MMNs than F1 contours for short-duration sweeps, whereas F1 sweep contours elicited larger MMNs than F0 contours for longer duration sweeps (i.e., 100 and 300 ms).

### MMN Peak Latency

**Figure 6:**
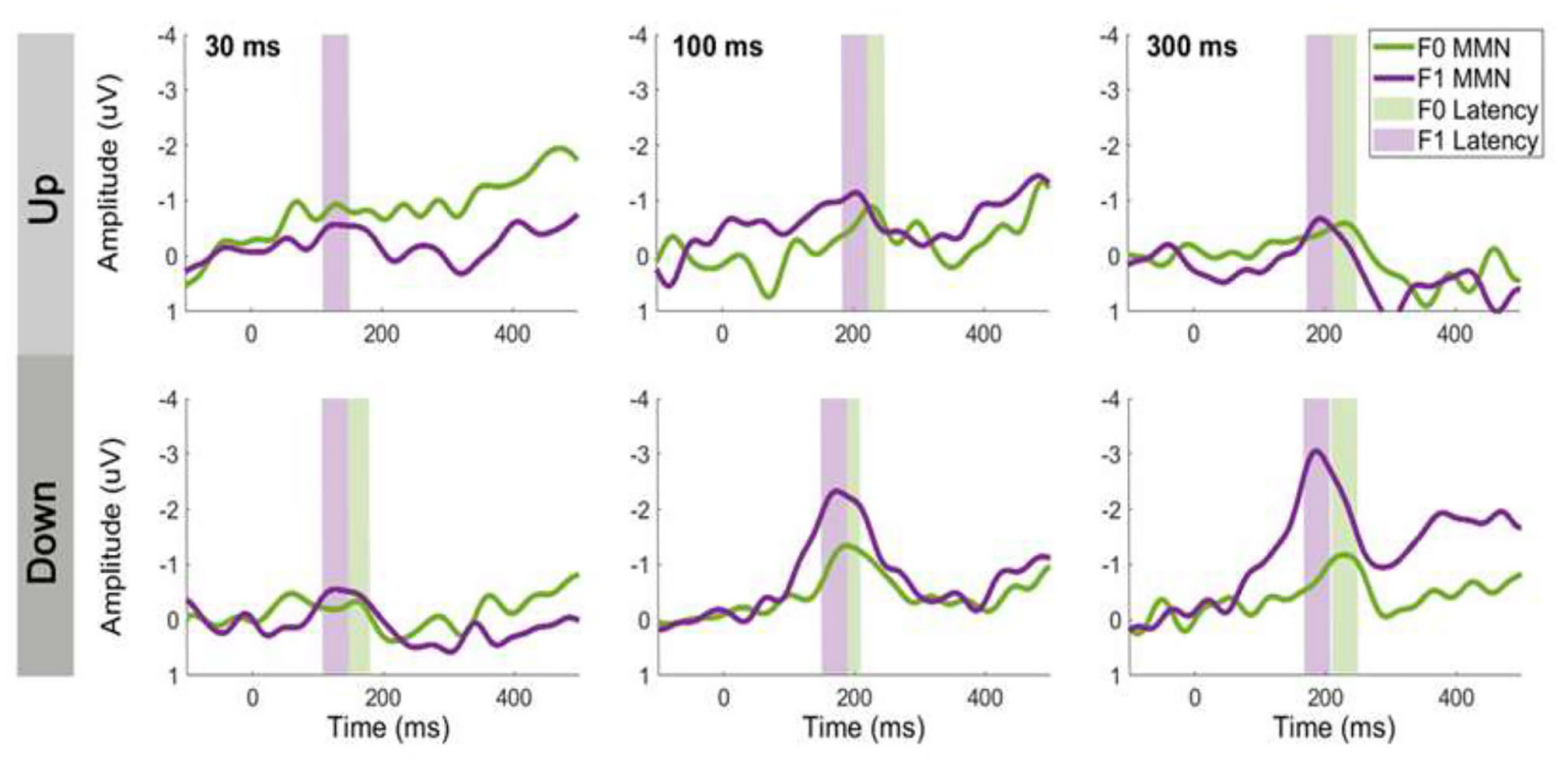
The effect of formant frequency on MMN latency for upward (top panels) and downward (bottom panels) tone sweeps. Left to right panels show sweeps at three timescales (30, 100, and 300 ms). Green and purple shaded region indicate peak latency ±20-ms window. MMN waveforms are recorded at electrode Fz and computed based on same-stimulus method.

Figure 6 shows the average MMN peak latency for upward (top panel) and downward (bottom panel) tone sweeps at the F0 and F1 frequencies over three timescales (i.e., 30, 100, and 300 ms). A 2 × 2 × 3 repeated-measures ANOVA was conducted on the MMN peak latency with the factors direction (up vs. down), frequency (F0 vs. F1), and duration (30, 100, and 300 ms). There was a significant main effect of F0/F1 frequency on MMN peak latency, *F*_(1,14)_ = 5.081, *p* < 0.05, with the MMN latency associated with F1 contours emerged earlier than those with F0 contours. There was no significant effect of temporal scale on tone sweep-elicited MMN latencies, *F*_(2,28)_ = 2.157, *p* = 0.135. The latencies of the MMN responses did not differ significantly between upward and downward frequency sweeps, *F*_(1,14)_ = 0.110, *p* = 0.745. Aside from the main effects, the analysis resulted in only one significant interaction, namely, between the tone sweep's direction and duration, *F*_(2,28)_ = 3.387, *p* < 0.05. Pair-wise comparisons using Bonferroni corrections (family wise α = 0.05) on F0/F1 contours at each condition revealed that the MMNs of F1 contours peaked significantly earlier than those evoked by F0 contours only in the 30-ms and 300-ms down sweep conditions, *t*_(14)_ = 2.180, *p* < 0.01 and *t*_(14)_ = 2.668, *p* < 0.01, respectively.

### MMN Topography: Lateralization Effect

**Figure 7:**
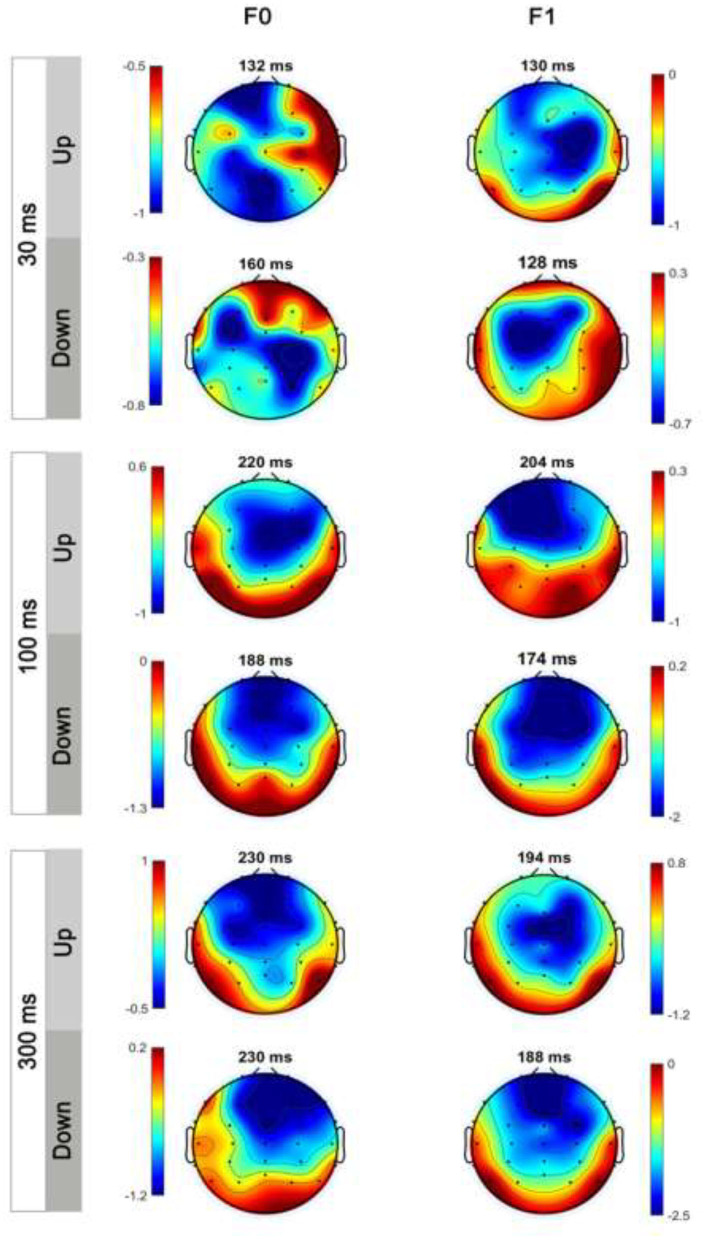
Scale distributions of the electroencephalogram (EEG) topography at MMN mean amplitude for tone sweeps in the F0 and F1 formant frequency for 30 ms (top panels), 100 ms (middle panels), and 300 ms (bottom panels). Color bar is scaled to the MMN mean amplitude in each topography. Negativity is indicated by blue and positivity by red color.

**Figure 8:**
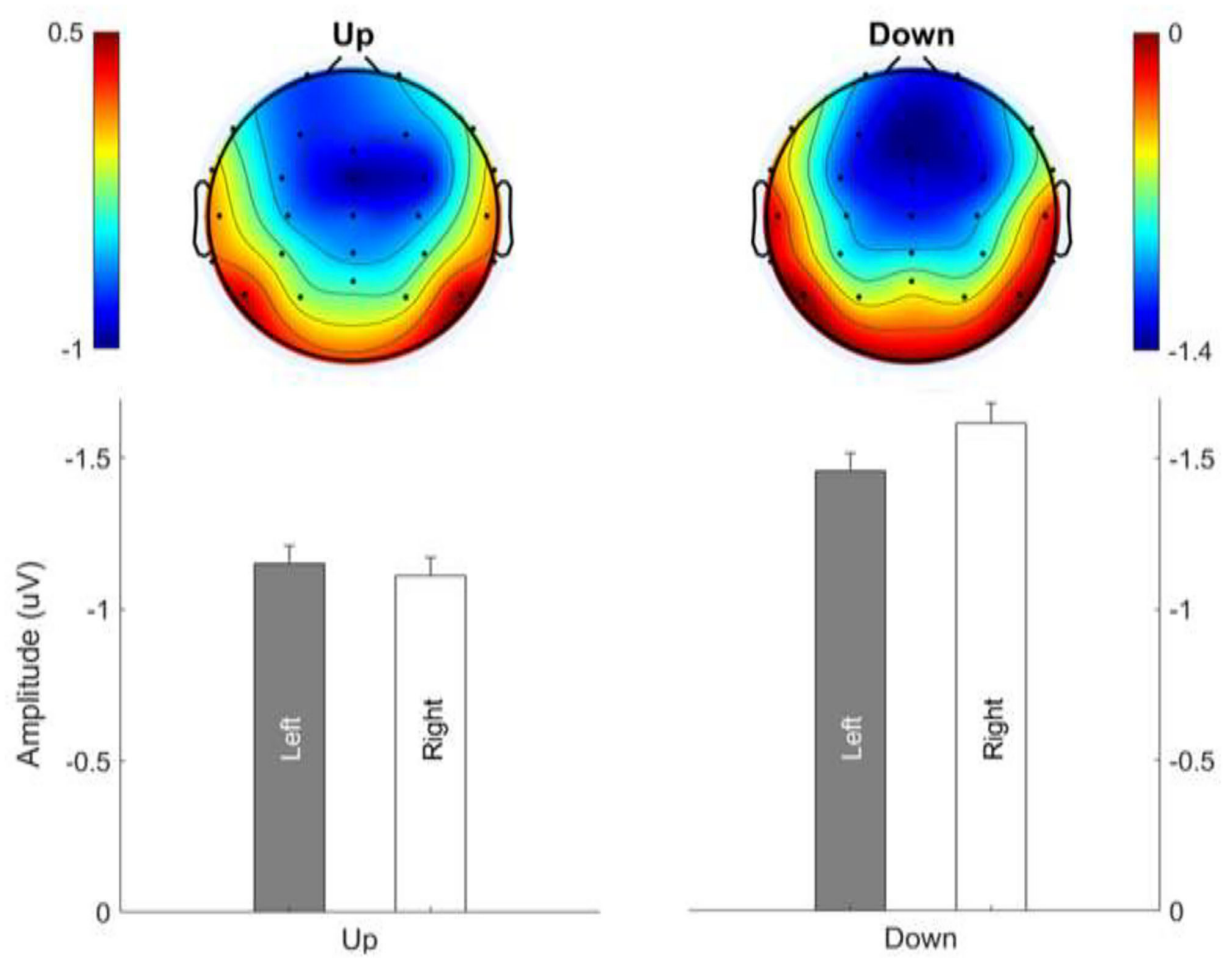
Hemispheric lateralization of MMN topography for upward and downward frequency sweeps. Color bar is scaled to the MMN mean amplitude in each topography. Negativity is indicated by blue and positivity by red color. Downward sweeps showed a right hemispheric dominance.

**Figure 9:**
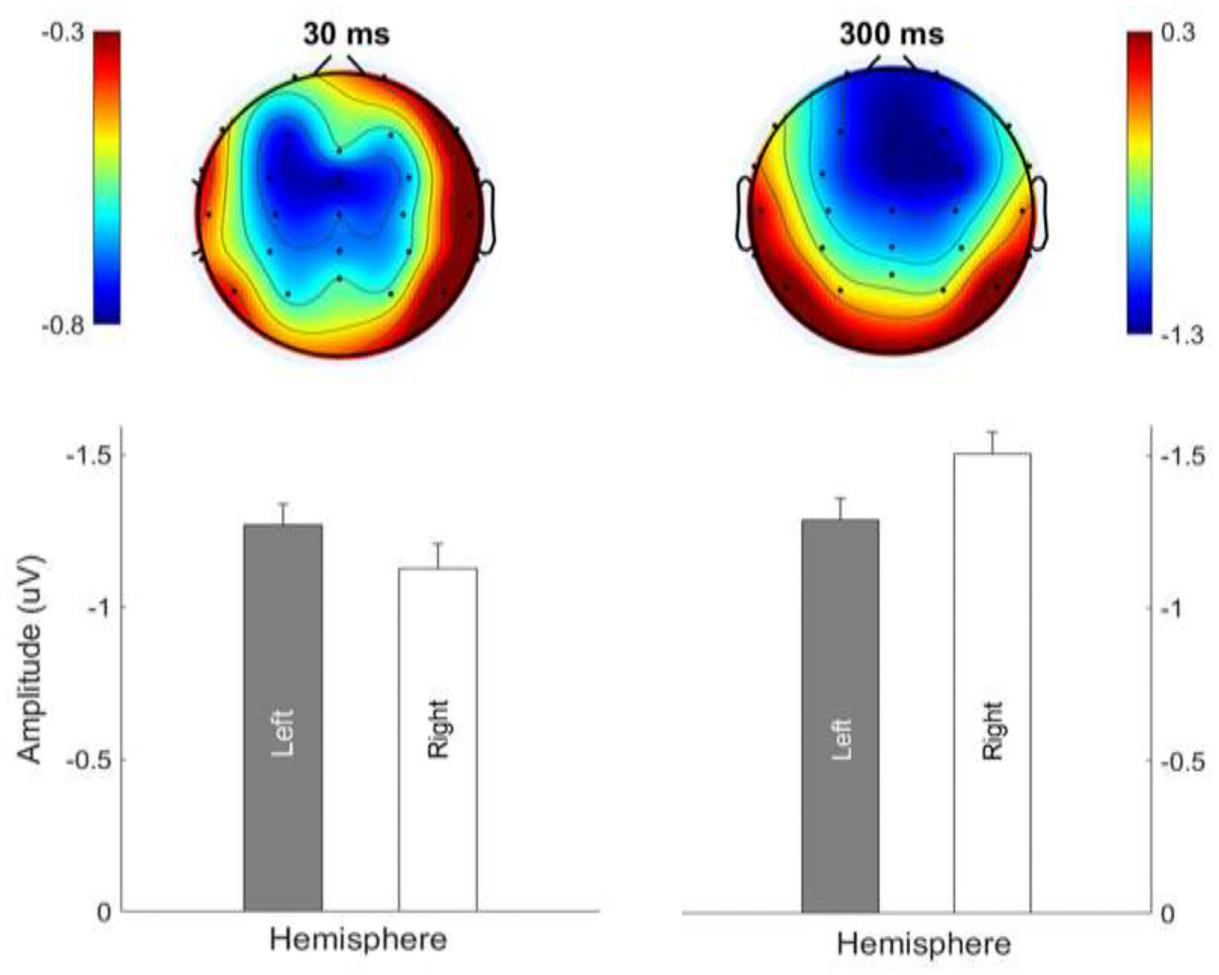
Hemispheric lateralization of MMN topography for frequency sweeps at local (30 ms; left panels) and global (300 ms; right panels) timescales. Color bar is scaled to the MMN mean amplitude in each topography. Negativity is indicated by blue and positivity by red color.

Figure 7 shows the average MMN topographies at the peak latency for all sweep conditions, which were dominated by frontocentral negativity. MMN topographies comparing hemispheric dominance for frequency sweeps at local and global timescales are shown in Figure 8. To test for the putative interhemispheric asymmetries for local and global time-scale sweeps, a three-way repeated-measures ANOVA with factors of hemisphere (left vs. right), sweep duration (30, 100, and 300 ms), and sweep direction (up vs. down) was performed. F0 and F1 frequency contours were combined for this analysis. There was a significant main effect of hemisphere on tone sweep-elicited MMN topographies, *F*_(1,209)_ = 5.175, *p* < 0.05. An effect of hemisphere was also revealed in a significant interaction between hemisphere and direction [*F*_(1,209)_ = 7.127, *p* < 0.01], as well as between hemisphere and duration [*F*_(2,418)_ = 14.291, *p* < 0.001]. Further *post-hoc* analysis on the interaction of lateralization and direction showed a significant right hemispheric dominance for downward sweep, *t*_(629)_ = 3.173, *p* < 0.01, but no significant hemispheric effect was found for upward sweeps (Figure 8). In addition, *post-hoc* analysis using Bonferroni corrections (family wise α = 0.05) on the interactive effect of lateralization and duration on MMN topography showed a significant left hemispheric dominance of MMN topography for the 30-ms sweep commensurate with local timescale, *t*_(419)_ = −2.083, *p* < 0.05. For longer duration sweeps (i.e., 300 ms) commensurate with global timescale, a significant right hemispheric lateralization effect was observed, *t*_(419)_ = 4.034, *p* < 0.001. No significant lateralization effect of MMN response was revealed for the 100-ms sweep condition. To illustrate the interhemispheric asymmetry, the mean MMN amplitudes over frontal central clusters in each hemisphere were separately plotted for local and global timescale frequency sweeps, as shown in Figure 9.

### Behavioral Results

**Figure 10:**
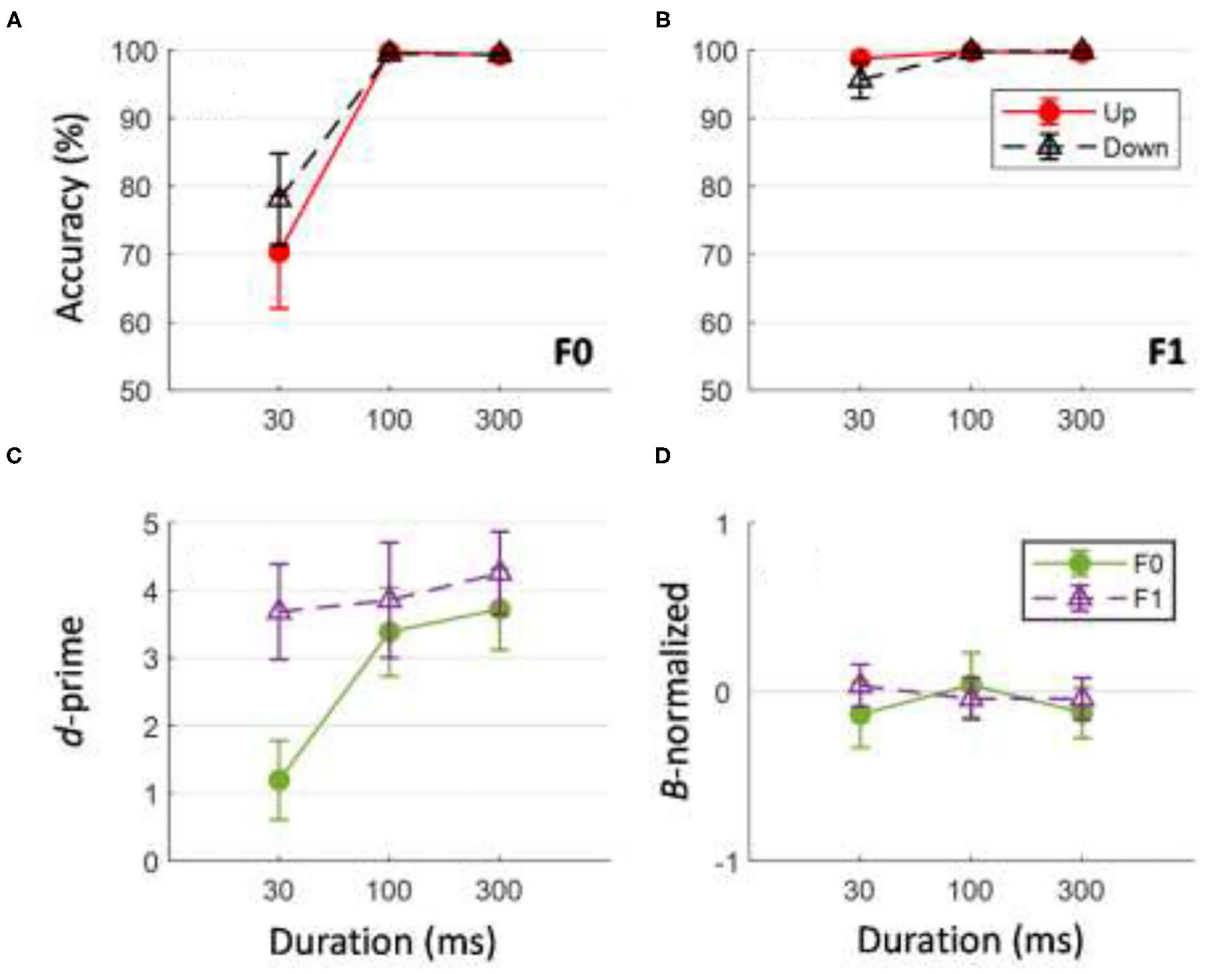
Behavioral accuracy and ß-normalized values in direction identification of tone sweeps for F0 **(A)** and F1 **(B)** frequency formants across all three durations. Bottom panel shows corresponding *d*-prime **(C)** and ß-normalized values **(D)** for behavioral performance. In **(D)**, value near 0 indicates no response bias in behavioral task. Error bars indicates ±1 standard error.

Figure 10 shows performance accuracy in identifying the direction of tone sweeps at different timescales for tone sweeps at the F0 (Figure 10A) and F1 (Figure 10B) formant frequencies. The corresponding *d*-prime and ß-normalized values for the behavioral task are illustrated in Figures 10C,D. A three-way repeated measures ANOVA on sweep-direction identification accuracy was performed with the factors frequency (F0 vs. F1), duration (30, 100, and 300 ms), and direction (up vs. down). There was a significant main effect of F0/F1 frequency on the accuracy of tone sweep direction identification, *F*_(1,11)_ = 39.707, *p* < 0.001. There was a significant main effect of tone sweep duration on the accuracy of tone sweep direction identification, *F*_(2,202)_ = 44.11, *p* < 0.001. Tone sweep direction did not have a significant effect on identification accuracy, *F*_(1,11)_ = 0.112, *p* = 0.744. The analysis yielded only one significant interaction of F0/F1 frequency and duration on accuracy of tone sweep identification, *F*_(2,22)_ = 38.052, *p* < 0.001. *Post-hoc* analysis using Bonferroni corrections (family wise α = 0.05) on duration effects showed a significant difference in identification accuracy only between tone sweep durations at 30 vs. 100 ms and 30 vs. 300 ms [*t*_(23)_ = −13.95 and −13.81, respectively; both *p* < 0.001]. Pairwise comparisons on F0/F1 frequencies at each duration indicated that at short timescales (30 ms), behavioral accuracy for identifying the F1 frequency formant was higher than that for sweeps modulated at F0, *t*_(23)_ = −4.374, *p* < 0.001. At a global timescale of 300 ms, the identification accuracy was not significantly different for tone sweeps modulated at F1 compared to those modulated at F0, *t*_(23)_ = −2.460, *p* = 0.022 (based on Bonferroni corrections family wise α = 0.05).

A two-way repeated measures ANOVA was conducted on *d*-prime values with frequency (F0 and F1) and duration (30, 100, and 300 ms) as factors. Upward and downward sweeps were combined for this analysis as direction did not generate a significant effect on identification accuracy (Figure 10C). Tone sweep identification at the F1 formant frequency resulted in larger *d*-prime values than those at the F0 formant frequency, *F*_(1,11)_ = 10.335, *p* < 0.05. The analysis also yielded a significant main effect of duration; longer duration tone sweeps resulted in larger *d*-prime values, *F*_(2,11)_ = 11.518, *p* < 0.001. A significant interaction of formant and duration was found, *F*_(2,22)_ = 6.472, *p* = 0.006. To see if subjects showed any key bias in the sweep-direction judgment task, ß-normalized values were analyzed with respect to tone sweep durations and F0/F1 frequency. Pair-wise *t*-test comparisons with “0” response (i.e., no bias) resulted in all non-significant results [all *t*_(11)_ < −1.2, all *p* > 0.191].

## Discussion

Using an auditory oddball paradigm, the present study provides electrophysiological evidence of an interaction between speech-relevant frequency regions and timescales in early cortical processing of frequency-modulated sweeps. First, our findings show that Mandarin Chinese speakers can implicitly, without attentional effort, selectively differentiate the direction of non-speech signals containing linguistically relevant FM features in certain formant frequency and timescale regions. The results showed clear MMNs evoked by all types of frequency sweeps modulated with F0 and F1 pitch contour at local and global timescales. Secondly, a significant interaction of MMN response pattern elicited by F0 and F1 formant frequency and global and local timescales was observed. Contrary to our predictions, a more pronounced MMN component was observed when the frequency sweep contours varied at F0 compared to the F1 formant at shorter time local timescale, whereas F1 sweep contours evoked larger MMN response than F0 contours at the global timescale. Consistent with previous reports, an asymmetrical MMN response pattern for implicit encoding of the direction of FM sweeps was observed with stronger MMN response associated with downward frequency sweeps. A right hemispheric dominance of MMN response amplitude was observed at the global timescale (300 ms) condition, consistent with the putative right hemispheric dominance for processing longer timescale acoustic features (Poeppel, 2003). Overall, our findings suggest that linguistically relevant timescale (more specifically local and global timescales) and formant frequency can interactively influence the pre-attentive processing of frequency sweeps.

### Interaction Between Timescale and Spectral Contour in FM-Sweep Processing

Importantly, our finding of the interaction between timescale and spectral contour suggests that these two dimensions, which are the two primary features of speech and music, are processed as an integrated unit, rather than independently, in early cortical stage of FM-sound processing. This is consistent with Zatorre and Gandour's (2008) integrated approach to speech processing, which proposes that low-level acoustic features are processed by the brain as a function of linguistic factors. Support for this view came from neuroimaging data showing that non-speech stimuli such as narrowband noise, pure tones, or tone sweeps emulating certain speech cues can, nevertheless, elicit the same cortical responses uniquely specific to speech signals (Joanisse and Gati, 2003; Zaehle et al., 2004; Boemio et al., 2005; Schönwiesner et al., 2005). Viewed in a broader context, the interrelation between pitch and temporal dimensions during sound processing has been studied extensively in the area of musical sequence processing at the attentive level. While inconsistencies still remain regarding whether pitch and time features are processed independently or interactively, one of the more well-known models, the model of dynamic attending, argue in favor of an integrated account (Large and Jones, 1999; Jones et al., 2002, 2006; Prince et al., 2009). The model is based on the notion that pitch and time dimensions interact together to form a meaningful unit in driving a listener's attention to future events. As speech perception is an intricately complex process that contains many levels of processing, relevant low-level acoustic features must be resolved and represented first before higher level encoding can occur (Zatorre and Gandour, 2008). The implication of this may be that by processing the spectral and temporal features of FM sounds in an interactive pattern at an early stage can drive a listener's attention toward certain sound features to facilitate subsequent stages of speech encoding. Future studies should examine conditions where FM features do not carry any linguistic information to see whether the pattern of neural responses may differ depending on the linguistic status of these low-level acoustic features.

### Processing FM-Sweep Spectral Contours

Consistent with the prevalence of downward falling tones in Mandarin Chinese, we also observe larger MMN response associated with frequency sweeps with falling pitch contour than frequency sweeps with rising pitch contours. This is in line with the higher occurrence of tone 4 (falling contour) compared to tone 2 (rising contour) reported for Mandarin Chinese (Xu, 1994; Xu et al., 2002; Liu et al., 2007). In addition, tone 4 has a larger pitch range than rising tones, which may contribute to the Mandarin listeners' differing responses to the rising and falling pitch contours (Xu et al., 2002). Behavioral studies have also indicated Mandarin Chinese speakers have better detection ability at downward FM sweeps and downward pitch contours in non-speech sounds (Bent et al., 2006; Luo et al., 2007; Giuliano et al., 2011). ERP studies using passive listening paradigm have also demonstrated larger MMN response when processing downward frequency sweeps compared to upward sweeps (Kung et al., 2020). The down preference pattern in FM-direction sensitivity has also been demonstrated in animal studies showing a higher percentage of down-preference neurons in inferior colliculus of pallid bats (Williams and Fuzessery, 2010) and similarly in cat's primary auditory cortex (Mendelson and Cynader, 1985), reflecting how the cortical system evolved to tune to species-typical communication (e.g., downward sweeps present in echolocation pulse) critical for survival. In terms of hemispheric lateralization, frequency sweeps with falling contours at both pitch height (F0 and F1) resulted in right hemispheric dominance, whereas rising frequency contours did not elicit hemispheric dominance. The right hemispheric lateralization associated with processing downward frequency sweeps is inconsistent with previous studies on lexical tone processing demonstrating a left hemispheric dominance for pitch contours (Wang et al., 2013). While Wang et al. (2013) showed a right hemispheric dominance when processing pitch height, the present study failed to obtain any hemispheric effect due to pitch height (i.e., F0 vs. F1) manipulation of frequency sweeps. This discrepancy may be due to the linguistic function of lexical tone stimuli used in Wang et al. (2013) that distinguishes itself from the non-speech frequency sweep materials used in the present study.

### Pre-attentive Processing of FM Sweeps at Different Timescales

The current result revealed that, in addition to disentangle speech signal's spectral-pitch variations, the human brain can pre-attentively process the different functional types of non-speech pitch variations that occur at different timescales. This suggests that the human brain can track both local and global-level pitch contours in the incoming non-speech acoustic signal (i.e., frequency sweep). This finding is consistent with previous psychophysical findings on FM-sweep processing, which has reported near-ceiling performance on sweep direction identification accuracy at the timescale close to local (30 ms) and global (160 and 320 ms) timescale ranges in Mandarin Chinese speakers (Luo et al., 2007). It was speculated that perceptual sensitivity to FM-sweep with lexical timescale may be transferred to perceptual sensitivity at other FM durations, including those shorter than the lexical-related timescale (Luo et al., 2007). Our MMN response yielded by local and global timescale FM sweeps lends further support to this notion. The finding with respect to processing different levels of timescales is also consistent with existing studies showing that unattended processing of speech pitch contours can be observed at the lexical-level tone and sentence-level pitch variations (Xi et al., 2010; Li and Chen, 2015). The main difference is that most of these studies have typically employed natural lexical-tonal materials modulated at relatively longer durations (i.e., 300–550 ms), whereas an acoustic variation free of lexical meaning with a shorter time local scale is employed in our study. Our findings add to current evidence by showing that non-speech frequency sweep contours, free of lexical meaning, may be processed at an early stage with reference to its linguistic function.

### Timescale and Lateralization of MMN Response

Apart from the interaction effects, our finding of higher left hemispheric MMN response associated with processing frequency sweeps at local timescale and higher right hemispheric dominance associated with frequency sweeps at the global timescale lends further support to the asymmetric sampling in time (AST) hypothesis [e.g., the two time-scale integration model (2TW1)] in speech processing (Poeppel, 2003; Luo and Poeppel, 2007; Poeppel et al., 2008; Teng et al., 2016). According to the AST model, the speech information or auditory signal is asymmetrically represented in the auditory areas according to time domain; auditory input with short (~20–40 ms) temporal windows is preferentially parsed and analyzed by the left auditory areas, whereas auditory information with temporal windows at a global scale (~150–250 ms) is preferentially extracted by the right hemisphere (Poeppel, 2003; Boemio et al., 2005; Luo and Poeppel, 2012). Our current observation of the hemispheric asymmetric MMN response with respect to the temporal scale of frequency sweeps is in line with the AST hypothesis, which has been receiving support by a growing body of neurophysiological evidence (Poeppel, 2003; Boemio et al., 2005; Morillon et al., 2010; Luo and Poeppel, 2012). In line with this, the differential lateralization in the brain for processing acoustic/speech signals over local and global timescales have also been demonstrated for frequency-modulated tones in a series to create local and global increasing/decreasing pitch patterns (Sanders and Poeppel, 2007), pitch discrimination of pure tones and frequency sweeps (Johnsrude et al., 2000; Brechmann et al., 2002; Meyer et al., 2002; Poeppel et al., 2004), as well as melodic contours with local or global pitch violation (Peretz, 1990; Schiavetto et al., 1999).

### Implications for Frequency Sweeps in Tone Language Processing

Our findings indicate that spectral contour and timescale interacts with each other in pre-attentive auditory processing of frequency sweeps with reference to its linguistic function. The MMN pattern suggests that Mandarin Chinese listener's perception could depend relatively more on F0 contours when processing local timescale information and relied more on F1 contours when resolving global timescale information. While this is contrary to our prediction, there has been some speculation that the different forms/degrees of syllable contractions often used in spontaneous speech in Mandarin Chinese could dramatically shorten the duration of one syllable. For example, the duration of two syllables in ran-hou (然後) is heavily reduced to 82 ms in spontaneous speech (Tseng, 2005). In addition, tones in Mandarin Chinese involves turning point that contains more than one directional information within the syllabic level. Therefore, the identification of sweep directions at a shorter timescale may be more crucial to tone language speakers. This possibility and its implication for tone language processing should be further examined.

Interestingly, frequency sweeps, while it constitutes the fundamental units in differentiating acoustic speech units, have no linguistic content implied. Why is a non-speech signal (i.e., frequency sweeps) processed at an early stage with reference to its linguistic function? We speculate that this may reflect the potential top-down effects of tone language on processing basic acoustic cue in speech. In tone languages that use highly constrained and parameterized frequency variations to make lexical distinctions, the ability to track the information changes in frequency sweeps becomes even more relevant biologically. The putative top-down effects is in line with previous behavioral studies demonstrating an advantage of tone language speakers in identifying non-speech pitch contours, click trains, or tone complex that resemble speech trajectories relevant to their native language experience (Francis et al., 2003; Bent et al., 2006; Luo et al., 2007; Xu and Pfingst, 2008; Cabrera et al., 2014, 2015) and in discriminating pure-tone pitch intervals (Giuliano et al., 2011). At the cortical level, our findings are also consistent with studies demonstrating earlier and larger ERP response when processing pure-tone pitch intervals or comparing Mandarin tone contrasts in tone language speakers (Chandrasekaran et al., 2009; Giuliano et al., 2011). Future studies should examine whether the interactive modulatory effects of spectral contour and timescale on pre-attentive processing of frequency sweeps are different for non-tone language speakers.

## Conclusion

In summary, our results showed that the brain can, in an unattended state, process dynamic changing frequency sweeps conveying linguistically functional formant pitch contour and timescale information. Results indicate that timescale and spectral contour interact with each other in pre-attentive auditory processing of frequency sweep signals. The MMN amplitudes showed a reversed pattern with respect to F0/F1 contours according to its timescale. An asymmetrical interhemispheric topology was observed with respect to processing local- and global-level frequency sweep contours, consistent with existing studies on the lateralization of encoding speech units of different timescales. Findings suggest that non-speech frequency sweep signal, free of lexical content, is processed at an early stage with reference to its linguistic function.

## Data Availability Statement

The raw data supporting the conclusions of this article will be made available by the authors, without undue reservation.

## Ethics Statement

The studies involving human participants were reviewed and approved by Research Ethics Committee of National Taiwan University, Taiwan. The patients/participants provided their written informed consent to participate in this study.

## Author Contributions

I-HH and W-TY designed the experiment and prepared the figures and interpreted the results. The auditory stimuli and experimental tasks were programmed by I-HH and W-TY. W-TY performed EEG data collection. The manuscript was written by I-HH. All authors contributed to data analysis.

## Conflict of Interest

The authors declare that the research was conducted in the absence of any commercial or financial relationships that could be construed as a potential conflict of interest.
