# Supplementary material for: The Interaction Between Timescale and Pitch Contour at Pre-attentive Processing of Frequency-Modulated Sweeps
Source: Front Psychol. 2021 Mar 23;12:637289. doi: 10.3389/fpsyg.2021.637289 (PMC8021897; doi:10.3389/fpsyg.2021.637289)
Supplement: Supplementary file 1 [file Table_1.DOCX]

**Supplementary File S1**

Post-hoc analysis on the three-way interaction (per family wise α= 0.05) indicated that the interaction between sweep duration and direction on MMN response was significant at the level of F1 contour, *F*(2, 28) = 6.840, *p* < 0.05, but failed to reached significance at the level of F0 contour, *F*(2, 28) = 2.122, *p* = 0.138. Further analysis at the F1 contour revealed that down sweeps, but not up sweeps, contributes to the significant effect, *F*(2, 28) = 13.465, *p* < 0.001. Specifically, pair-wise comparisons on the time scales of down sweeps showed that the significant effect lies mainly in the comparisons between 30 vs. 100 ms and 30 vs. 300 ms sweeps (*t*(14)=3.982, *p* < 0.001 and *t*(14)=4.455, *p* < 0.001, respectively). The interaction between F0/F1 contour and duration on MMN response was not significant for both upward and downward sweeps, (*F*(2, 28) = 1.594, *p* = 0.22 and *F*(2, 28) = 2.395, *p* = 0.109, respectively). The interaction between frequency and direction of tone sweeps did not vary across the three levels of time scales, (*F*(2, 28) = 0.325, *p* = 0.725, *F*(2, 28) = 0.784, *p* = 0.109, *F*(2, 28) = 1.235, *p* = 0.306 for 30, 100 and 300 ms-sweeps, respectively).
